# Screening Patterns of Nonalcoholic Fatty Liver Disease in Children with Obesity in Canadian Primary Care: A Cross-Sectional Study

**DOI:** 10.1155/2022/8435581

**Published:** 2022-12-24

**Authors:** Rachael Morkem, Rebecca Theal, David Barber, Jennifer Flemming, John Queenan, Mohit Kehar

**Affiliations:** ^1^Department of Family Medicine, Queen's University, Kingston, Ontario, Canada; ^2^Departments of Medicine and Public Health Sciences, Queen's University, Kingston, Ontario, Canada; ^3^Division of Pediatric Gastroenterology, Hepatology and Nutrition, Children's Hospital of Eastern Ontario, University of Ottawa, Ottawa, Ontario, Canada

## Abstract

**Background:**

Nonalcoholic fatty liver disease (NAFLD) is the most common pediatric chronic liver disease, and children with a body mass index (BMI) ≥95th percentile are recommended to be screened for NAFLD by liver enzymes.

**Objectives:**

This study aimed to determine the frequency and predictors of screening for NAFLD among children with obesity in Canada and to evaluate a sample of children with suspected NAFLD.

**Methods:**

This cross-sectional study used data from the Canadian Primary Care Sentinel Surveillance Network, a repository of electronic medical record data from Canadian primary care practices.

**Results:**

Of *n* = 110,827 children aged 9–18 years, 13.9% (*n* = 9,888) had a BMI ≥95^th^ percentile. Only 8.7% (*n* = 859) of these patients were screened for NAFLD in the last year, and 23.6% (*n* = 2336) were ever screened. Using logistic regression, screening in the last year was associated with demographic and clinical characteristics, including previous liver enzyme assessment, prior antidiabetic prescription, and prior anxiolytic prescription. Among children with suspected NAFLD (*n* = 1,046), 34.7% had a BMI ≥99^th^ percentile and approximately 8% were at increased risk of significant liver disease.

**Conclusion:**

The study revealed low screening rates for NAFLD in Canadian primary care and highlighted the important role of primary care providers in identifying and managing pediatric NAFLD.

## 1. Introduction

Nonalcoholic fatty liver disease (NAFLD) is the most common cause of chronic liver disease in children [1]. The estimated global prevalence of NAFLD is ∼7.6% in the general pediatric population and upward of 30% in children with obesity [1]. The global prevalence of NAFLD among adults is increasing and is estimated to be 23–25% [2, 3]. In Canada, the frequency of adults living with NAFLD is expected to increase by 20% through 2030 and is projected to be the leading cause of cirrhosis in young adults by 2040 in Canada [4, 5]. Similar concerning trends are seen in children, with data showing a rapid increase in the prevalence of NAFLD over the past 2 decades [6, 7]. Currently, there is no epidemiological data on rates of pediatric NAFLD in Canada. However, obesity, a leading risk factor for NAFLD, has been rising steadily in Canadian children in recent decades [8].

It has been recommended that children at high risk for NAFLD (including obesity) be screened for NAFLD [9], and in 2017, the North American Society for Pediatric Gastroenterology, Hepatology and Nutrition (NASPGHAN) published a guideline recommending alanine transaminase (ALT) to screen for NAFLD in children aged 9 years and older with a body mass index (BMI) ≥95^th^ percentile [10]. To confirm the diagnosis of NAFLD, children with chronically elevated liver enzymes and other causes of hepatic steatosis must be excluded. Liver biopsies should be considered in children at increased risk of nonalcoholic steatohepatitis (NASH) and/or advanced fibrosis [10]. Although a minority of those with NAFLD (3% to 5%) develop clinically significant liver disease, NAFLD is predicted to be the leading indication for liver transplantation in young adults over the next decade [11]. In children, the natural history of NAFLD is limited, but studies have shown that children may have a more aggressive phenotype [10, 12], with over 50% of pediatric patients with NAFLD having some form of hepatic fibrosis by the age of 11–13 years [13]. Suffice it to say that NAFLD has been reported as early as 2 years of age, with NASH-related cirrhosis as early as 8 years of age [13, 14]. In a recent Swedish study, compared to matched controls, children and young adults with NAFLD had higher rates of overall, cancer-, liver-, and cardio-metabolic-related mortality[15]. Overall, there is a significant need for ongoing assessment and follow-up for children at risk for NAFLD and progressive liver disease.

Primary care providers (PCPs) are the first contact point with the healthcare system, and they play a vital role in the identification, treatment, specialist referral, and follow-up of children with NAFLD. Despite this, the literature suggests that PCPs tend to have limited awareness of the prevalence, scope, and management of NAFLD in adults [16, 17]. The results of a recent study of a small group of PCP practices revealed that most primary care practitioners were unfamiliar with pediatric NAFLD [18]. The data regarding PCPs' screening practices for pediatric NAFLD are lacking on a national scale, and the epidemiology remains largely unknown in Canada. It is essential to understand the current screening pattern in Canada, as well as the predictors of screening, in order to improve the care for children with NAFLD.

The information held within primary care electronic medical records is extremely valuable to uncover this knowledge gap and determine the burden and current screening practices of obesity-associated NAFLD in Canada. The objectives of this study were to determine the frequency of pediatric NAFLD screening and its predictors at a national level using population representative data and describe a baseline cohort of patients identified with suspected NAFLD.

## 2. Methods

### 2.1. Study Design and Database

This is a cross-sectional cohort study that used national primary care data from the Canadian Primary Care Sentinel Surveillance Network (CPCSSN). CPCCSN is a national network comprising eleven practice-based research networks that collect and amalgamate ongoing clinical data from primary care electronic medical records on over 1.5 million patients across Alberta, British Columbia, Quebec, Manitoba, Newfoundland and Labrador, Nova Scotia, and Ontario. This repository of deidentified data contains a breadth of longitudinal data, including demographics, diagnoses, procedures, exam measures, laboratory test results, prescriptions, and referrals. The data resource has been described elsewhere [19]. The study was reviewed for ethical compliance by the Queen's University Health Sciences and Affiliated Teaching Hospitals Research Ethics Board (PAED-505-20).

### 2.2. Study Population

The study population consisted of children aged 9–18 years with a BMI-for-age ≥95^th^ percentile, that had visited their PCP between January 1, 2018, and December 31, 2019 (a two-year contact group). In accordance with the recommendations of the NASPGHAN, the age of 9 years and older was chosen for the screening of children with obesity (BMI-for-age ≥95^th^ percentile) [10], and a two-year contact group has been shown to be a useful approach for estimating the population size of the practice [20]. Only patients with a documented sex and birth year were included in the study population.

### 2.3. Demographic and Clinical Characteristics

Patient age was determined as of December 31, 2019. Rural or urban patient locations were determined based on the second digit of the postal code, which indicates whether the patient lives in an urban [1–9] or rural area (0), as defined by Canada Post delivery areas.

A patient's weight status was categorized using BMI-for-age cut points recognized by the World Health Organization and the Centre for Disease Control [21]. These categories are defined as underweight ≤5^th^ percentile, normal or healthy weight from the 5^th^ percentile to <85^th^ percentile, overweight ≥85^th^ to <95^th^ percentile, and obese as ≥95^th^ percentile. The last height and weight measurements, recorded in the electronic medical record on the same day, were used to derive the BMI for age and determine the percentile. These measurements, along with birth month and year to determine an exact age at the time of measurement, were used to determine BMI for age using a SAS Macro provided by the Centre for Disease Control. Any patient with a BMI-for-age ≥95^th^ percentile was considered eligible for screening according to NASPGHAN guidelines [10].

Using validated algorithms based on billing, diagnosis, medication, and lab data, the following comorbid health conditions were identified: diabetes, hypertension, dyslipidemia, attention deficit hyperactivity disorder, and depression [22–24]. In addition, we included two conditions for which there was no validated definition: polycystic ovarian syndrome, and sleep apnea. These patients were identified by searching the database for International Classification of Diseases, Version 9 (ICD-9) codes 256 and 327, respectively.

Anatomical Therapeutic Chemical Classification Codes were used to identify the use of the following medication classes: antipsychotics (N05A), anxiolytics (N05B), antidepressants (N06A), psychostimulants (N06B), antihypertensives (C02), and antidiabetics (A10). A patient was classified as being on the listed medication class if the patient received at least one prescription for that class in the year preceding their last visit in 2018 or 2019. Healthcare utilization was assessed by counting the number of visits in the previous year. Specialist referrals and alcohol status for each patient were assessed based on any documentation within the CPCSSN database.

### 2.4. Screening and Identification of Potential NAFLD

Prior laboratory liver panel tests were evaluated for the presence of lab ALT and/or aspartate aminotransferase (AST). NAFLD screening was defined by the presence of a record of an ALT test in the past year. ALT at any time in the data was also recorded. Suspected NAFLD was defined in one of two ways: (1) Elevated age and sex-specific ALT levels (22 units per litre (U/L) of blood for girls and 26 U/L for boys) in the absence of chronic viral hepatitis (*B* or *C*) (ICD-9 code 070), no documentation of alcohol use, hepatotoxic medications (defined by ATC codes C01BD01, amiodarone; H02, corticosteroids; L01BA01, methotrexate; J05A, highly active antiretroviral therapy; N03AG01, valproic acid), Wilson's disease, or autoimmune hepatitis (ICD-9 code 275.1 or 571.42); (2) prior diagnosis of NAFLD determined by ICD-9 code, 571.5, 571.8, and 571.9.

### 2.5. Statistical Analysis

We characterized the basic demographic and clinical characteristics of the study population (patients eligible for NAFLD screening and patients with suspected NALFD) using descriptive statistics including frequencies. The clinical and demographic characteristics of patients who were eligible and screened for pediatric NAFLD in the last year were compared with those who were eligible and not screened, using crude odds ratios to test for significant differences. Multiple logistic regression was conducted to explore the independent association between demographic and clinical characteristics and screening for NAFLD, including age (9–12 years vs 13–18 years), sex (male vs. female), location (rural vs. urban), BMI (obese vs. severe obesity), comorbid conditions (yes vs. no), prior medications (yes vs. no), and prior liver panel (yes vs. no). Adjusted odds ratios with 95% CIs were calculated. Missing data were excluded from the model. All analyses were performed in SAS statistical software, version 9.6.

## 3. Results

### 3.1. Study Participants and Screening Cohort

**Figure 1:**
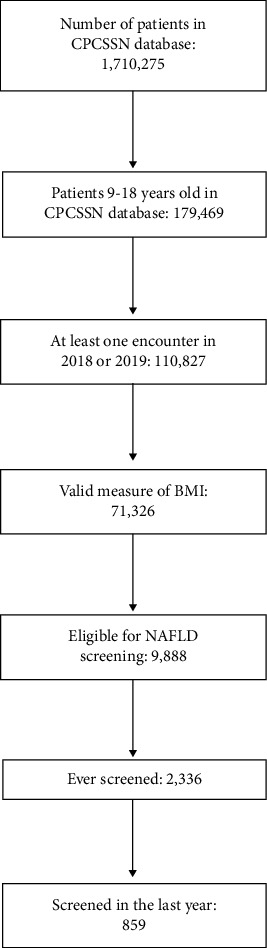
Selection of study population.

There were 110,827 pediatric patients, aged 9 to 18 years, who had an encounter with a CPCSSN-participating PCP between 2018 and 2019. Approximately two-thirds (*n* = 71,326, 64.4%) of these patients had a valid measure of BMI. In total, 9,888 children had a BMI ≥95^th^ percentile and were eligible for NAFLD screening during the study period (Figure 1). Overall, patients who were eligible for NAFLD screening tended to be older (13–18 years vs. 9–12 years; 59.9%) and male (55.3%). The majority of patients were from urban locations (80.5%) and in the 95^th^–98^th^ BMI percentile (vs ≥99^th^; 78.1%). Preexisting comorbid conditions (0.24–8.4%), prior medication use (0.6–11.6%), and prior specialist referral (0.9–13.1%) were identified in a smaller proportion of patients. The most common comorbid condition was depression/anxiety, and correspondingly, psychotropics, including antidepressants, anxiolytics, and antipsychotics, were the most commonly prescribed medications. The characteristics of all patients eligible for screening are further described in Table 1.

### 3.2. Screening Rate and Factors Associated with NAFLD Screening

Among the eligible population (*n* = 9,888), only 8.7% were screened for NAFLD via ALT within the last year and only 23.6% were ever screened. In comparison to patients who were eligible and not screened, patients who were screened for NAFLD in the last year were more likely to be older (13–18 vs 9–12), female, from urban locations (vs rural), and severely obese (vs obese). In addition, patients who were screened tended to have more comorbid conditions, prior medications, prior AST/ALT testing, and increased healthcare utilization. Comparisons of patients grouped by NAFLD screening status and unadjusted ORs are further described in Table 2.

### 3.3. Multivariate Analysis for Factors Associated with Screening in the Past Year

The multivariate regression analysis revealed that prior liver panels (ALT and/or AST testing) had the strongest association with NAFLD screening in the past year when controlling for potential confounders. Patients with prior liver panel testing had 2.94 times higher odds (95% CI 2.49–3.48) of getting screened than those who had never had a liver panel. In addition, patients had higher odds of being screened if they were prescribed anxiolytic and/or antidiabetic medication, older (13–18 vs 9–12), female, and living in an urban setting (vs rural) and had a higher BMI (≥99^th^ vs 95^th^–98^th^ percentile) (Table 2). Outcomes did not significantly differ after excluding patients prescribed anxiolytics, antidepressants, and/or antipsychotics (Supplementary Table 1).

### 3.4. NAFLD Cohort

**Figure 2:**
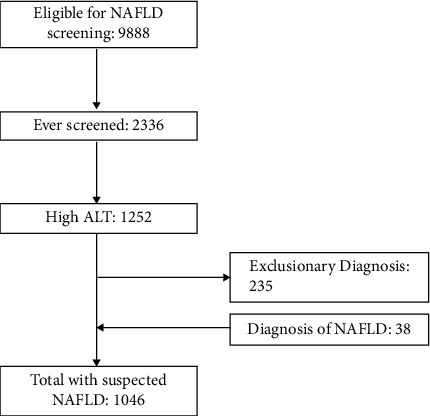
Identification of children with suspected NAFLD.

Of the 2,336 patients who had ever been screened for NAFLD, over 50% had elevated age and sex-specific ALT. After excluding other aetiologies and adding patients with an ICD-9-coded NAFLD diagnosis, a total of 1,046 patients with suspected NAFLD were identified (Figure 2). Patients with suspected NAFLD had a median ALT of 31 U/L (interquartile range = 18) and approximately 8% of patients had an ALT >80 U/L. Patients with suspected NAFLD were more often older (13–18 vs 9–12 years old) and male and from an urban location. Approximately one-fifth of patients had comorbid dyslipidemia (17.1%), attention deficit hyperactivity disorder (17.1%), and depression (22.9%). A smaller proportion had diabetes (5.6%), hypertension (9.1%), polycystic ovarian syndrome (1.7%), and sleep apnea (0.86%). Antidepressants were the most prescribed medication (26.6%), followed by psychostimulants (16%) and antipsychotics (9.4%). Less than one-third of patients with suspected NAFLD had documentation of a prior specialist referral to gastroenterology, pediatrics, or endocrinology. On average, patients visited their PCPs 4 times in the past year. Characteristics of patients with suspected NAFLD are further described in Table 3.

## 4. Discussion

In our study, we examined the proportion of childhood NAFLD screening in a large national multicenter database and identified very low screening rates (8.7% in the past year and 23.6% ever screened). Among the children with suspected NAFLD, many had associated comorbidities, including depression, hypertension, and dyslipidemia. As a result of its silent nature, it is likely that patients with NASH cirrhosis may develop the disease as youths and remain undiagnosed until they have reached adulthood [25], highlighting the importance of early detection and intervention. There is a critical need for PCPs and pediatric specialists to work together toward establishing a clinical referral pathway to address the identified gaps in screening and management of pediatric NAFLD in Canadian primary care.

In this study, less than nine percent of patients eligible for screening had their ALT evaluated in the past year and less than one-quarter of patients had ever been evaluated. Previous research has been limited regarding screening rates and PCP-based management of pediatric NAFLD, and there are no available data in the context of Canadian primary care. The only study conducted on a national level to assess screening patterns for pediatric NAFLD was conducted in the United States, which found that 54.0% of children with obesity and 24.0% of children who were overweight had been screened for NAFLD [26]. In comparison with this study, our study shows significantly lower screening rates. It is unclear why Canadian screening rates are so different than in the United States; however, variations in Canadian and US health systems may play a role. In addition, knowledge gaps surrounding pediatric NAFLD may be a significant factor in the low screening rates identified in our study. Studies assessing adult-specific NAFLD management practices by clinicians have identified significant gaps in the understanding and care of NAFLD [16, 17]. Based on a recent Canadian study, few primary care physicians are aware of pediatric NAFLD [18]. Our study points out the urgency of developing strategies to improve pediatric NAFLD awareness, including a dedicated continuing medical education program and Canadian specific guidelines.

Along with PCP knowledge and management practices, there are many factors that may contribute to the disparity between eligible children who were screened versus those who were not screened for NAFLD. The regression model revealed that children with prior liver panels were significantly more likely to be screened for NAFLD in the last year. This may indicate that certain patients are undergoing regular liver monitoring in line with the American Association of Pediatrics and NASPGHAN clinical guidelines [9, 10]. Similarly, children were more likely to be screened for NAFLD if they had previously been prescribed anxiolytics or antidiabetics. Ongoing liver monitoring is recommended with certain medications and may explain the relationship between prior prescriptions and screening. In the case of antidiabetics, the increased likelihood of NAFLD screening may also be attributed to the known link between NAFLD and type 2 diabetes. Type 2 diabetes is a significant risk factor for NAFLD, with an estimated 58% of patients with diabetes estimated to have NAFLD [3, 27, 28]. Interestingly, preexisting diabetes was not a significant predictor of NAFLD screening in this cohort. However, this may imply that screening for NAFLD or ALT testing is more likely when diabetes treatment is required (i.e., more advanced disease or drug-related liver monitoring) versus simply a diagnosis of diabetes. This is supported by the finding that, of the antidiabetic medications prescribed to children in our cohort, 38% were for metformin, a drug which has guidelines to monitor ALT levels [29]. As such, this may account for part of the strong association between antidiabetic medications and screening in our study.

Additional predictors of NAFLD screening included being female, older age, having a higher BMI, and living in an urban setting. Unsurprisingly, we found that patients with NAFLD risk factors (i.e., older age and higher BMI) are more likely to be screened for NAFLD [3, 27, 30]. However, not all predictors can be explained with this reasoning. There were significantly more female patients screened for NAFLD than male patients, despite a higher proportion of male patients in the total eligible population as previously described [26]. Although the relationship between NAFLD and sex is complex, male sex is a commonly reported risk factor for NAFLD [3, 27, 30]. Specifically, in pediatric populations, adolescent males have been found to be at significantly increased risk for NAFLD compared to females [30, 31]. By understanding who is being screened and why, educational initiatives and quality improvement initiatives can be tailored to boost screening rates of pediatric NAFLD.

In addition to reporting screening rates, this study characterized the largest Canadian sample population to date with suspected pediatric NAFLD. Nearly 50% of patients ever screened for NAFLD had elevated age- and sex-specific ALT indicative of suspected NAFLD, with a median ALT of 31 U/L. Although not directly comparable to the true prevalence of pediatric NAFLD in Canada, this is in line with the current literature, which estimates the prevalence of NAFLD to be upwards of 30% among children with obesity [1]. Approximately 8% of patients with suspected NAFLD had ALT levels associated with an increased risk of significant liver disease and NASH (>80 U/L) [10, 32]. Most patients with suspected NAFLD were also male, obese, and older, which concurs with previous reports on adolescent NAFLD populations [26, 30, 31]. Comorbid attention deficit hyperactivity disorder, depression, diabetes, dyslipidemia, and hypertension were prevalent in patients with suspected NAFLD (5.6–22.9%). The rates of dyslipidemia and hypertension were slightly higher than in previous reports [26, 31], while the rate of comorbid diabetes was similar to a study in children with biopsy-proven NAFLD in the United States [33]. In a recent meta-analysis, the prevalence of depression in adult NAFLD patients was 18.2%, compared to 22.9% in the current study [34]. Overall, the characteristics of patients with suspected NAFLD in this study are consistent with the existing literature. The high levels of comorbid disease highlight the need for screening and disease monitoring in pediatric NAFLD patients.

In 2020, international experts called for a new definition of NAFLD. The idea is to use the term metabolic dysfunction-associated fatty liver disease rather than NAFLD and make it a positive diagnosis rather than an exclusionary one. Although this more appropriate and useful definition has been met with mostly positive responses in adults, it may have a tangled path to follow in pediatrics. Current guidelines recommend that a child with an elevated ALT level be evaluated further to confirm a diagnosis of NAFLD, which requires excluding other causes of steatosis. This would likely require a referral to a specialist. In our study, only one-third of patients with suspected NAFLD had a specialist referral to gastroenterology, endocrinology, or pediatrics. Consequently, improved referral patterns are needed to diagnose and treat patients with NAFLD, particularly those with multiple risk factors or signs of a more severe disease. A recent retrospective chart review of pediatric patients with suspected NAFLD revealed a high proportion of patients with bloodwork suggesting an alternative diagnosis. However, comprehensive testing was infrequently performed, highlighting the importance of maintaining a differential diagnosis amongst children presumed to have NAFLD [34], especially to ensure appropriate treatment is initiated. Referral pathways have been developed for PCPs who treat adults with NAFLD with the objective of improving knowledge of these conditions as well as increasing detection and treatment of advanced cases [35]. It is imperative to create such a referral pathway for pediatric patients in order to improve patient referrals and initiate early treatment. Although there is no currently approved pharmaceutical NAFLD treatment, preclinical and clinical trials are underway [36]. In addition, with increased understanding of the pathogenesis of NAFLD, many efforts are being made toward developing targeted interventions for NAFLD [37]. The future of therapeutic landscape in NAFLD looks promising. A strength of this study is that it is the first to analyze a large cohort of suspected pediatric NAFLD cases in Canada and to assess factors associated with screening for pediatric NAFLD in children with obesity at a national scale. By systematically collecting large population data over time, CPCSSN data are positioned to monitor trends in service use, understand current screening practices of children with obesity for disease, and help inform the burden of NAFLD in an at-risk population. However, several limitations should be considered when interpreting our study results. About 36% of active primary care patients aged 9–18 did not have a documented BMI measurement, although previous research with CPCSSN data implies that missing BMI measurements may be more likely to be within the normal weight range [38, 39]. A retrospective administrative database may exhibit misclassification bias, and data from CPCSSN are limited by the quality of the electronic medical record data sources, rarely include unstructured fields such as chart notes, and may overlook other potential predictors of pediatric NAFLD screening. As well, we do not have data on histology, which is the gold standard for diagnosing NAFLD, so we cannot indicate the proportion of patients who fall along the spectrum of NAFLD. Similarly, we do not have data on ultrasonographic examination, so we cannot comment on rates of all methods of NAFLD screening. Although ultrasound is not recommended for pediatric NAFLD screening [10], it is a readily available and noninvasive tool used for detecting hepatic steatosis and may have been performed in place of ALT testing. There is also a lack of data related to ethnicity, which has previously been associated with the development of NAFLD [40, 41]. Finally, our data come from a Canadian population and might not be representative of other areas of the world.

We report on the current practices of screening for pediatric NAFLD in children with obesity in primary care in comparison to the established recommendations in our study. Our study assessed the predictive factors for screening and characterized the patients who were identified as having suspected pediatric NAFLD. The screening rate of eligible pediatric patients for NAFLD is low in contrast to NASPGHAN guidelines. This likely highlights a significant gap in knowledge surrounding pediatric NAFLD management in Canadian primary care. Only heightened awareness amongst PCPs and close connections between primary care and pediatric specialists may decrease the number of unscreened or undiagnosed pediatric patients and improve the ongoing management of pediatric NAFLD. This study will contribute to the body of knowledge and help inform the gaps so future recommendations can be made, including ways of implementing screening measures in real-life primary health care. Future studies linking primary care and administrative data may be required to investigate disease prevalence and investigate the care trajectory of patients with suspected NAFLD.

## Figures and Tables

**Table 1:** Eligible NAFLD screening population.

Characteristics	Total eligible, *n*(%) (*N* = 9888)
Age, years	
9–12	3967 (40.1)
13–18	5921 (59.9)

Sex	
Male	5465 (55.3)
Female	4423 (44.7)

Location	
Rural	1887 (19.5)
Urban	7803 (80.5)
Missing	198

BMI, percentile	
≥95^th^–<99^th^ (obese)	7658 (77.5)
≥99^th^ (severe obesity)	2230 (22.6)

Preexisting conditions	
Diabetes	90 (0.91)
Hypertension	215 (2.2)
Dyslipidemia	272 (2.8)
Depression/Anxiety	835 (8.4)
PCOS	24 (0.24)

Prior medications	
Antipsychotics	272 (2.8)
Anxiolytics	188 (1.9)
Antidepressants	1147 (11.6)
Antihypertensives	127 (1.3)
Antidiabetics	62 (0.60)

Prior liver panel	
AST and/or ALT	1685 (17.0)

**Prior primary healthcare utilization, one year average**	**3.99 (3.96)^†^**

Prior specialist referral	
Gastroenterology	85 (0.9)
Endocrinology	119 (1.2)
Pediatrics	1296 (13.1)

^†^Reported as mean (standard deviation); BMI, body mass index; PCOS; polycystic ovarian syndrome; AST, aspartate aminotransferase; ALT, alanine transaminase.

**Table 2:** Comparison of eligible patients aged 9–18 years screened and not screened for NAFLD in the last year.

Characteristics	Screening in the last year			
	No, *n* (%) (*N* = 9029)	Yes, *n* (%) (*N* = 859)	Unadjusted odds ratio (95% CI)	Adjusted odds ratio^†^ (95% CI)
Age, years				
9–12	3747 (41.5)	220 (25.6)	Ref	Ref
13–18	5282 (58.5)	639 (74.4)	2.06 (1.76–2.41)	1.63 (1.38–1.93)

Sex				
Male	5056 (56.0)	409 (47.6)	Ref	Ref
Female	3973 (44.0)	450 (52.4)	1.4 (1.22–1.61)	1.32 (1.14–1.54)

Location				
Rural	1757 (19.8)	130 (15.6)	Ref	Ref
Urban	7099 (80.2)	704 (84.4)	1.34 (1.1–1.63)	1.37 (1.12–1.67)
Missing	173	25		

BMI, percentile				
≥95^th^–<99^th^ (obese)	7049 (78.1)	609 (70.9)	Ref	Ref
≥99^th^ (severe obesity)	1980 (21.9)	250 (29.1)	1.46 (1.25–1.71)	1.36 (1.15–1.60)

Preexisting conditions^‡^				
Diabetes	63 (0.70)	27 (3.1)	4.62 (2.93–7.29)	1.56 (0.87–2.80)
Hypertension	173 (1.9)	42 (4.9)	2.63 (1.87–3.72)	1.35 (0.89–2.04)
Dyslipidemia	218 (2.4)	54 (6.3)	2.71 (1.99–3.67)	0.99 (0.70–1.42)
Depression/Anxiety	521 (5.8)	81 (9.4)	2.58 (2.08–3.19)	1.20 (0.91–1.57)
PCOS	17 (0.19)	7 (0.81)	4.36 (1.80–10.53)	1.41 (0.51–3.93)

Prior medications^‡^				
Antipsychotics	233 (2.5)	49 (5.7)	2.39 (1.74–3.28)	1.09 (0.74–1.60)
Anxiolytics	148 (1.6)	40 (4.7)	2.93 (2.05–4.19)	1.99 (1.34–2.97)
Antidepressants	970 (10.7)	177 (20.6)	2.16 (1.80–2.58)	1.14 (0.91–1.44)
Antihypertensives	111 (1.2)	16 (1.9)	1.52 (0.89–2.59)	0.65 (0.34–1.28)
Antidiabetics	36 (0.40)	26 (3.0)	7.80 (4.69–12.99)	2.88 (1.54–5.38)

Prior liver panel^‡^				
AST and/or ALT	1338 (14.8)	347 (40.4)	3.90 (3.36–4.52)	2.94 (2.49–3.48)

Prior primary healthcare utilization 1 year average	3.82 (3.8)^§^	5.6 (5.3)^§^	1.08 (1.07–1.10)	1.02 (1.01–1.04)

Prior specialist referral^‡^				
Gastroenterology	67 (0.74)	18 (2.1)	2.87 (1.69–4.84)	1.53 (0.88–2.69)
Endocrinology	96 (1.1)	23 (2.7)	2.56 (1.63–4.06)	1.13 (0.68–1.88)
Pediatrics	1154 (12.8)	142 (16.5)	1.35 (1.12–1.63)	1.11 (0.90–1.36)

^†^ Multivariate logistic regression (fully adjusted) model; ^‡^yes vs no (reference); ^§^reported as mean (standard deviation); BMI, body mass index; PCOS; polycystic ovarian syndrome; AST, aspartate aminotransferase; ALT, alanine transaminase.

**Table 3:** Patients with suspected NAFLD.

Characteristics	Total suspected NAFLD *n* (%) (*N* = 1046)
Age, years	
9–12	189 (18.1)
13–18	857 (81.9)

Sex, % male	542 (51.8)

Location	
Rural	246 (24.0)
Urban	781 (76.0)
Missing	19

BMI, percentile	
≥85^th^ (underweight to normal)	7 (0.67)
86^th^–94^th^ (overweight)	4 (0.38)
≥95^th^–<99^th^ (obese)	667 (63.8)
≥99^th^ (severe obesity)	363 (34.7)
Missing	5

ALT (*n* = 1021)	
Median ALT	31 (18)^†^
ALT >40	335 (32.3)
ALT >80	82 (7.8)

Health conditions	
Diabetes	59 (5.64)
Hypertension	95 (9.1)
Dyslipidemia	179 (17.1)
ADHD	179 (17.1)
Depression	239 (22.9)
PCOS	18 (1.7)
Sleep apnea	9 (0.86)

**Medications**	
Antipsychotics	98 (9.4)
Anxiolytics	77 (7.36)
Antidepressants	278 (26.6)
Psychostimulants	167 (16.0)
Antihypertensives	55 (5.3)
Antidiabetics	42 (4.0)

Prior primary healthcare utilization over 1 year	4 (6)^†^

Prior specialist referral	
Gastroenterology	34 (3.25%)
Pediatrics	231 (22.1%)
Endocrinology	53 (5.07%)
Weight clinic/dietician	2 (0.19%)

^†^Reported as median (interquartile range); BMI, body mass index; ALT, alanine transaminase; ADHD, attention deficit hyperactivity disorder; PCOS, polycystic ovarian syndrome.

## Data Availability

The datasets generated and analyzed during the present study are available from the corresponding author upon reasonable request. Data from the Canadian Primary Care Sentinel Surveillance Network (CPCSSN) may be released upon application to the CPCSSN Data Access Committee, who can be contacted via https://cpcssn.ca/dar/.

## Supplementary Materials

Supplementary Materials Supplementary materials include one table describing multivariate logistic regression models with and without patients prescribed anxiolytics, antipsychotics, and/or antidepressants.
