# Supplementary material for: Screening Patterns of Nonalcoholic Fatty Liver Disease in Children with Obesity in Canadian Primary Care: A Cross-Sectional Study
Source: Can J Gastroenterol Hepatol. 2022 Dec 24;2022:8435581. doi: 10.1155/2022/8435581 (PMC9805392; doi:10.1155/2022/8435581)
Supplement: Supplementary Materials — include one table describing multivariate logistic regression models with and without patients prescribed anxiolytics, antipsychotics, and/or antidepressants. [file 8435581.f1.docx]

Supplementary Table 1. Comparison of multivariate logistic regression models with and without patients prescribed anxiolytics, antipsychotics and/or antidepressants

|  | Including anxiolytics, antipsychotics and/or antidepressants | Excluding anxiolytics, antipsychotics and/or antidepressants |
| --- | --- | --- |
| Characteristics | Adjusted Odds Ratio †  (95% CI) | Adjusted Odds Ratio †  (95% CI) |
| Age, years  9-12  13-18 | Ref  1.63 (1.38-1.93) | Ref  1.55 (1.29-1.85) |
| Sex  Male  Female | Ref  1.32 (1.14-1.54) | Ref  1.34 (1.13-1.59) |
| Location  Rural  Urban | Ref  1.37 (1.12-1.67) | Ref  1.43 (1.13-1.80) |
| BMI, percentile  ≥95^th^-<99^th^ (obese)  ≥99^th^ (severe obesity) | Ref  1.36 (1.15-1.60) | Ref  1.37 (1.13-1.65) |
| Pre-existing Conditions ‡  Diabetes  Hypertension  Dyslipidemia  Depression/Anxiety  PCOS | 1.56 (0.87-2.80)  1.35 (0.89-2.04)  0.99 (0.70-1.42)  1.20 (0.91-1.57)  1.41 (0.51-3.93) | 1.71 (0.86-3.40)  0.96 (0.53-1.73)  1.02 (0.68-1.54)  1.30 (0.86-1.97)  1.60 (0.49-5.28) |
| Prior Medications ‡  Antipsychotics  Anxiolytics  Antidepressants  Antihypertensives  Antidiabetics | 1.09 (0.74-1.60)  1.99 (1.34-2.97)  1.14 (0.91-1.44)  0.65 (0.34-1.28)  2.88 (1.54-5.38) | -  -  -  0.87 (0.32-2.37)  4.25 (1.97-9.16) |
| Prior Liver Panel ‡  AST and/or ALT | 2.94 (2.49-3.48) | 3.31 (2.74-4.00) |
| Prior Primary Healthcare utilization 1 Year Average | 1.02 (1.01-1.04) | 1.03 (1.01-1.05) |
| Prior Specialist Referral ‡  Gastroenterology  Endocrinology  Pediatrics | 1.53 (0.88-2.69)  1.13 (0.68-1.88)  1.11 (0.90-1.36) | 2.26 (1.24-4.10)  1.24 (0.70-2.18)  1.15 (0.91-1.45) |

† Multivariate logistic regression (fully adjusted) model; ‡Yes vs no (reference); §Reported as Mean (Standard Deviation); BMI, Body Mass Index; PCOS; Polycystic Ovarian Syndrome; AST, aspartate aminotransferase; ALT, alanine transaminase.
